# Bio-Based and Solvent-Free Epoxy Vitrimers Based on Dynamic Imine Bonds with High Mechanical Performance

**DOI:** 10.3390/polym17050571

**Published:** 2025-02-21

**Authors:** Lei Chen, Na Ning, Gang Zhou, Yan Li, Shicheng Feng, Zhengyan Guo, Yi Wei

**Affiliations:** Center for Civil Aviation Composites, Donghua University, 2999 North Renmin Road, Shanghai 201620, China; chenlei2946@163.com (L.C.); foreverluck7@sina.com (N.N.); 1215042@mail.dhu.edu.cn (G.Z.); 1235040@mail.dhu.edu.cn (Y.L.); fsctcu@163.com (S.F.); zyguo712@163.com (Z.G.)

**Keywords:** vanillin, bio-based aldehyde, epoxy vitrimers, dynamic imine bond, degradatility, reprocessability

## Abstract

Conventional epoxy thermosets, with irreversible crosslinking networks, cannot be reprocessed and recycled. Furthermore, the utilization of petroleum-based materials accelerates the depletion of non-renewable resources. The introduction of dynamic covalent bonds and the use of bio-based materials for thermosets can effectively address the above issues. Herein, a series of bio-based epoxy vitrimers with dynamic covalent imine bonds were synthesized via a simple solvent-free, one-pot method using vanillin-derived aldehyde monomers, 4,4-diaminodiphenylsulfone (DDS) and bisphenol F diglycidyl ether (BFDGE) as raw materials. The effect of crosslinking density, crosslinking structure and imine bond content on the resulting bio-based vitrimers was studied, demonstrating their excellent thermal properties, UV shielding and solvent resistance, as well as outstanding mechanical properties compared to those of the previously reported vitrimers. In particular, the cured neat resin of vitrimer had a maximum tensile strength of 109 MPa and Young’s modulus of 6257 MPa, which are higher than those of previously reported imine-based vitrimers. The dynamic imine bonds endow these vitrimers with good reprocessability upon heating (over 70% recovery) and degradation under acidic conditions, enabling recycling by physical routes and gentle degradation by chemical routes. This study demonstrates a simple and effective process to prepare high-performance bio-based and recycled epoxy thermosets.

## 1. Introduction

Epoxy resins exhibit excellent mechanical strength, stability and electrical insulation properties, which are widely used in composite materials, coatings and other fields [1,2,3]. However, conventional epoxy crosslinked networks are permanent once cured, thus hindering their ability to be repaired, reprocessed and recycled [4,5,6]. By introducing dynamic covalent bonds, it is possible to form covalent adaptable networks (CANs) that can break or reform in response to stimuli such as stress, temperature, radiation, solvents, etc. CANs perform reversible de-polymerization/dissociative processes or exchange/associative reactions, making the thermosets reusable, reprocessable, weldable, rectifiable and recyclable [6,7,8,9,10].

Vitrimer is a new type of thermoset material incorporating CANs, which, unlike conventional thermosets, exhibit thermoplastic behavior under specific conditions. Due to the dynamic exchange properties of CANs, vitrimers have the ductility and processability of a thermoplastic while maintaining the integrity of the thermoset network at operating or service temperatures [11,12,13,14]. A number of dynamic covalent bonds have been utilized to synthesize vitrimers, including ester [15,16,17], disulfide [18,19,20], acetal [21,22], imine [23,24], carbonate [25,26] and acylhydrazone [27,28] bonds. Among these dynamic bonds, imine bond is active at lower temperatures, and the transamination reaction is catalyst-free [29], providing superior reprocessing characteristics. The synthetic routes for imine vitrimers typically involve either the direct polycondensation of multifunctional aldehydes and amines [30,31,32] or the incorporation of imine bonds into a thermoset precursor [33,34,35], which subsequently undergoes crosslinking. However, compared to conventional epoxy resins, these vitrimers typically exhibit low thermal and mechanical properties [36,37], and poor solvent and chemical resistance, thus limiting their industrial utilization. Moreover, most reported imine vitrimers have come from non-renewable resources, reducing their sustainability [38,39]. Therefore, the development of bio-based epoxy vitrimers with excellent mechanical properties provides significant value in terms of environment protection and circular economy.

Vanillin is an important monoaromatic phenol produced at an industrial scale from lignin, and it is naturally abundant and biodegradable [40,41]. With its rigid aromatic ring, vanillin can endow the derived polymers with excellent mechanical properties [42], thermal stability [43] and chemical resistance [44]. Vanillin has reactive hydroxyl and aldehyde groups that can be diversely designed to meet the specific needs of material applications. Therefore, vanillin has been investigated for bio-based vitrimers [45,46,47,48]. Liu et al. [49] used vanillin and m-xylylenediamine to synthesize the curing agent Van-OH with adynamic imine bond. Van-OH and BFDGE were subsequently cured to obtain Van-EP bio-based vitrimers. The obtained imine epoxy vitrimers were remodelable, self-healable and degradable. However, their method of firstly synthesizing the imine-curing agent, making it undergo separation and purification processes and then subsequently using it to cure the epoxy resins involved multiple steps, and resulted in high costs and low yields, making it less attractive for industrial applications. Liang et al. [50] developed a simpler process using a facile one-pot method to prepare imine epoxy vitrimers, in which all reactants were dissolved in acetonitrile solvent, followed by the removal of the solvent and subjecting the mixture to curing. The inclusion of solvents caused potential hazards and increased processing costs. As a result, it remains challenging to prepare high-performance, solvent-free, and environmentally and process-friendly imine epoxy vitrimers.

To come up with a simple and practical process to produce imine epoxy vitrimers without the aforementioned issues, while simultaneously utilizing bio-based raw materials, a series of vanillin derivatives—including aliphatic dialdehydes (*di*-Ali), aromatic dialdehydes (*di*-Aro), and aromatic trialdehydes (*tri*-Aro)—were designed using vanillin as a raw material. Furthermore, a true one-pot process was developed that utilized the inherent low viscosity of the raw materials, thus eliminating the use of solvents. The bio-based imine epoxy vitrimers were easily prepared by mixing vanillin-derived aldehyde monomers, the DDS curing agent and commercially available BFDGE epoxy. The resins can be directly used to prepare the composite prepreg or used for composite infusion processes. The effects of different imine bond contents as well as different crosslinking network structures on vitrimers were systematically investigated. Additionally, their mechanical properties, stress relaxation, thermo-mechanical properties and solvent resistance performance were evaluated. It is also worth mentioning that this work presents the first use of *tri*-Aro and chain vitrimers (*di*-CHV) synthesized from *di*-Ali in the production of imine epoxy vitrimers. The synthesized vitrimers exhibited impressive mechanical properties, up to 109 MPa tensile strength and a Young’s modulus of 6257 MPa for the neat resin. Thanks to the presence of dynamic imine bonds, the prepared vitrimer materials were demonstrated to be degradable and reprocessable. This new approach, the use of a solvent-free, one-pot method, provides the potential for the large-scale, low-cost industrial production of robust, recyclable and degradable bio-based epoxy resin materials for fiber-reinforced composites.

## 2. Materials and Methods

### 2.1. Raw Materials

Bisphenol F diglycidyl ether (99%, BFDGE, NPEF-170, EEW = 170) was provided by Nan Ya Plastic (Kunshan, China) Co., Ltd. Glutaryl chloride (95%, GLC), terephthaloyl chloride (99%, TEC), 1,3,5-benzenetricarbonyl chloride (98%, BEC), 4,4-diaminodiphenyl sulfone (97%, DDS), and ethylenediamine (EDA) were purchased from Adamas-beta (Shanghai, China) Co., Ltd. Vanillin (99.5%), dimethyl sulfoxide (DMSO) and triethylamine (TEA) were obtained from Shanghai Meryer Chemical Technology (Shanghai, China) Co., Ltd. Sodium hydroxide (NaOH), ethyl acetate (EAC) and hydrochloric acid (HCl) were supplied by Shanghai Titan Technology (Shanghai, China) Co., Ltd. N,N-dimethylformamide (DMF), tetrahydrofuran (THF), acetone (ACE), and ethyl alcohol (EtOH) were obtained from Shanghai Aladdin Biochemical Technology (Shanghai, China) Co., Ltd.

### 2.2. Sample Preparation

**Synthesis of Bio-based Aldehyde Monomers.** Vanillin (0.3 mol) was added to a 100 mL NaOH (0.31 mol) aqueous solution and stirred to obtain a vanillin sodium salt solution. BEC (0.1 mol) was dissolved in EAC (120 mL) and slowly added dropwise to the vanillin solution at 0 °C. The reaction was carried out for 2 h at 0 °C, followed by 4 h at 50 °C. After the reaction was completed, the resulting white solid product was first washed with 4% NaOH aqueous solution, followed by three washes with 100 mL of distilled water (DI water). The aromatic trinaldehyde monomer (denoted as *tri*-Aro) was dried in a vacuum oven at 100 °C for 5 h. The aromatic dialdehyde monomer (denoted as *di*-Aro) was prepared from TEC by the same process.

Vanillin (0.1 mol) and TEA (0.1 mol) were dissolved in EAC (100 mL) solution at room temperature (RT). GLC (0.05 mol) was added dropwise to the above solution within 30 min at 5 °C and kept at RT for 24 h. The obtained by-product, triethylamine hydrochloride, was in solid form and was removed by filtration. The aliphatic dialdehyde monomer (denoted as *di*-Ali) was obtained after drying the solution via rotary evaporation.

**Preparation of Bio-based Imine Epoxy Vitrimers.** The bio-based aldehyde monomers prepared as mentioned above were mixed with BFDGE and dissolved under stirring at 110 °C. DDS was then added at 110 °C until it was completely dissolved. The solution was poured into a metal mold, cured at 180 °C for 2 h, and further cured at 200 °C for 1 h to yield the bio-based polyimine vitrimer cast. The chain vitrimer obtained by the bio-based aldehyde monomer *di*-Ali was named *di*-CHV. The phenyl vitrimers obtained by the bio-based aldehyde monomers *di*-Aro and *tri*-Aro were named *di*-PHV and *tri*-PHV, respectively.

In order to explore the effect of imine bond contents on the properties, we synthesized *di*-CHV with $\frac{n_{-CHO}}{n_{-CHO}+n_{-CH(O)CH2}}$ concentrations of 10%, 20%, and 30%, resulting in the corresponding samples named di-CHV-10, di-CHV-20, and di-CHV-30, respectively. Similarly, vitrimers with the same imine content were prepared for *di*-PHV and *tri*-PHV. Specific vitrimers and their components are shown in Table S1. As a comparative sample, BFDGE was cured directly by DDS (denoted as the control) without dynamic imine bonds in the crosslinked network.

## 3. Results

### 3.1. Synthesis of Biobased Imine Epoxy Vitrimers

#### 3.1.1. Synthesis of Vanillin-Based Aldehyde Monomers

**Scheme 1:**
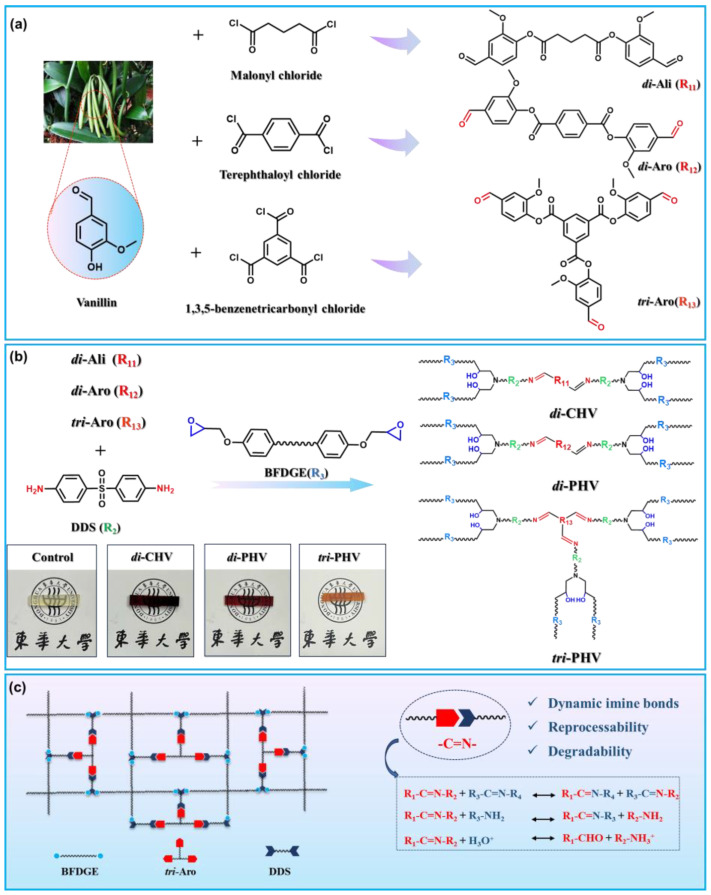
Synthetic route of (**a**) vanillin-based aldehyde monomers (*di*-Ali, *di*-Aro, and *tri*-Aro) and (**b**) imine vitrimer (*di*-CHV, *di*-PHV, *tri*-PHV); (**c**) dynamic crosslink of imine epoxy vitrimers.

The synthesis of imine epoxy vitrimers is illustrated in Scheme 1. Initially, *tri*-Aro, a vanillin derivative featuring three aldehyde groups, was prepared by combining vanillin with BEC. Similar methods were employed for the synthesis of *di*-Aro and *di*-Ali (Scheme 1a). The structure of vanillin-based aldehyde monomers *tri*-Aro, *di*-Aro and *di*-Ali was characterized by ^1^H NMR spectroscopy (Figure S1). In the ^1^H NMR spectrum of *di*-Ali, peaks at 2.8 ppm and 2.3 ppm were generated by α and β carbon protons of the ester group on the alkyl chain, confirming the incorporation of the aliphatic chain (Figure S1a). In the ^1^H NMR spectra of all vanillin-based aldehyde monomers, the peak at 7.5 ppm originates from the aromatic protons of the benzene ring in vanillin, confirming the incorporation of vanillin. Additionally, the peaks at 8.3 ppm (*di*-PHV) and 9.0 ppm (*tri*-PHV) originate from the aromatic protons of the benzene rings in terephthaloyl chloride and 1,3,5-benzenetricarbonyl chloride, respectively. Furthermore, the peak intensity at 10 ppm facilitated the determination of the number of aldehyde groups, confirming the successful synthesis of vanillin-based aldehyde monomers with varied structures. Through this step, multiple crosslinking sites (two or three aldehyde groups) were introduced, which was a pre-requisite for vitrimer synthesis.

#### 3.1.2. Synthesis of Vanillin-Based Imine Epoxy Vitrimers

The vanillin-based aldehyde monomers obtained above were reacted with DDS and BFDGE in a one-pot method to synthesize a range of imine epoxy vitrimers with diverse crosslink structures. The chemical structure was determined by FT-IR (Figure S2). As shown in Figure S2a, the characteristic peaks of the aldehyde group at 1682 cm^−1^ and ester group at 1750 cm^−1^ indicate the successful synthesis of vanillin-based aldehyde monomers. As shown in Figure S2b, the characteristic peaks of the amino group in DDS at 3332 cm^−1^ and 3362 cm^−1^, the aldehyde group in *tri*-Aro at 1682 cm^−1^, and the epoxy group in BFDGE at 910 cm^−1^ disappeared. A new characteristic peak at 1636 cm^−1^, attributed to the imine bond in *tri*-PHV, appeared, confirming the formation of imine bonds. Similarly, the IR spectra of *di*-PHV and *di*-CHV in Figure S2c exhibited the same characteristic peaks, indicating the successful synthesis of the target imine epoxy vitrimers. For comparison, this characteristic peak was absent in the control sample.

The influence of these crosslinked networks on thermodynamic and mechanical properties was systematically investigated. Furthermore, owing to the dynamic nature of imine bonding, we evaluated their vitrimeric behavior and achieved both recycling by physical routes and gentle degradation by chemical routes.

### 3.2. Thermal Properties of Bio-Based Imine Epoxy Vitrimers

**Figure 1:**
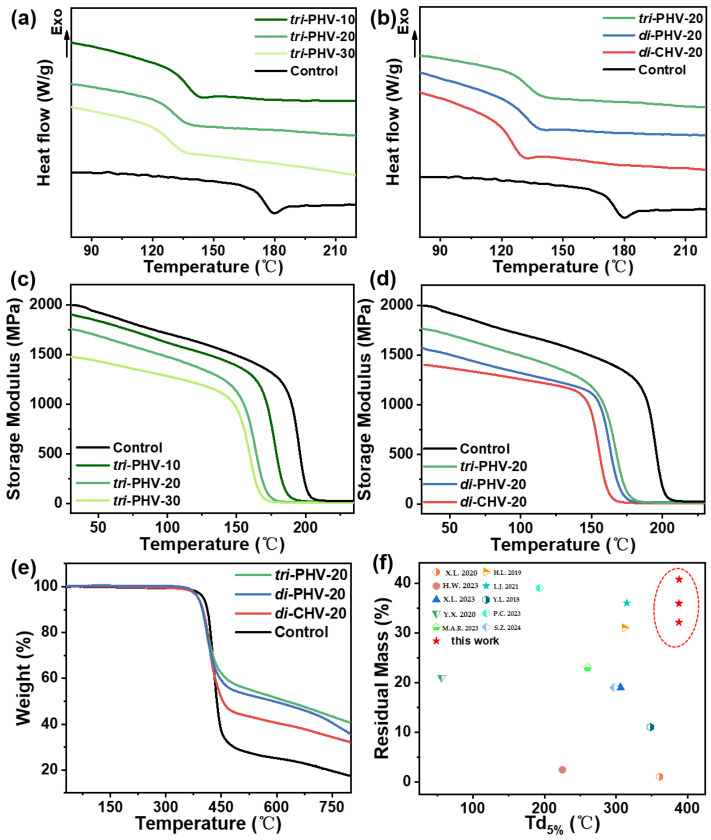
(**a**) DSC curves and (**c**) storage modulus of the control and *tri*-PHV with different imine bond contents; (**b**) DSC curves and (**d**) storage modulus of the control, *tri*-PHV-20, *di*-PHV-20 and *di*-CHV-20; (**e**) TGA curvesof the control, *tri*-PHV-20, *di*-PHV-20 and *di*-CHV-20 with different imine bond contents; (**f**) comparison of thermal stability of *tri*-PHV-20, *di*-PHV-20, and *di*-CHV-20 (in dash circle) with reference [31,33,34,37,40,43,46,49,50,51].

The glass transition temperature (*T_g_*), thermal–mechanical properties, and thermal stability of the imine epoxy vitrimers, obtained as described in the experimental section, were characterized using DSC, DMA, and TGA. The results are presented in Table 1 and Figure 1. Firstly, the effect of imine bond content on the thermodynamic properties of vitrimers is discussed, taking *tri*-PHV as an example for analysis. As shown in Figure 1a, the *T_g_* determined by DSC for *tri*-PHV-10, which contained 10% imine bonds, was 145.1 °C. As the dynamic imine bond content increased, the *T_g_* gradually decreased from 132.8 °C (*tri*-PHV-20) to 128.8 °C (*tri*-PHV-30). Similar trends can be observed for *di*-PHV and *di*-CHV (Figure S3b). This reduction in *T_g_* can be attributed to two main factors: firstly, the decrease in *T_g_* can be primarily ascribed to the reduction in the number of permanent crosslinks, which were replaced by dynamic imine bonds. This replacement increased the mobility of the polymer chains, leading to a lower *T_g_*. Secondly, the higher proportion of imine bonds accelerated the metathesis of the C=N bonds and the rearrangement of the network topology at a high temperature. In addition, the different crosslinked network structures also affected the *T_g_*. Specifically speaking, the *T_g_* of *di*-CHV was lower than that of *di*-PHV and *tri*-PHV with corresponding imine content (Figure 1b and Figure S3a). This disparity arose from the presence of flexible aliphatic chains in *di*-CHV rather than the rigid benzene rings in *di*-PHV and *tri*-PHV, resulting in the lowest *T_g_*. It was expected that *tri*-PHV, with its greater number of benzene ring substituents compared to that in *di*-PHV, would have a higher crosslinking density and *T_g_*. However, the experimental results indicate that the *T_g_* values of *tri*-PHV and *di*-PHV are close to each other. This observation may be attributed to the incomplete reaction of the three aldehyde substituents due to steric hindrance or the formation of cyclic oligomers [51]. Furthermore, the control without a dynamic bond and with the highest crosslinking density was more stable at elevated temperatures, resulting in a higher *T_g_* (173.2 °C) than that of the imine epoxy vitrimers.

The *T_g_* values obtained from DMA measurements were consistent with those from the DSC. As depicted in Figure 1c, *tri*-PHV undergoes a transition from a glassy to a rubbery state with increasing temperature [18]. The effect of imine bonding content on the thermodynamic properties is further demonstrated using *tri*-PHV as an example. For *tri*-PHV, the *T_g_* measured by DMA decreased progressively from 168.3 °C (*tri*-PHV-10) to 150.7 °C (*tri*-PHV-30) as the proportion of dynamic bonds increased (from 10% to 30%) (Figure 1c and Figure S3b). This decrease is attributed to the reduction in overall crosslinking density, as the introduction of dynamic imine bonds replaced some of the permanent crosslinks, increasing molecular mobility. Similar trends were observed in *di*-PHV and *di*-CHV. This can be explained by the fact that as the proportion of imine bonds increases, the reduced crosslinking density of the vitrimers, along with the accelerated metathesis of C=N bonds, leads to a decrease in *T_g_*. Similarly, the effect of the crosslinked structure on the thermodynamic properties should be fully considered. As depicted in Figure 1d, *di*-CHV exhibits lower *T_g_* values compared to *di*-PHV and *tri*-PHV, with the corresponding imine contents, due to the less rigid aliphatic chains in the crosslinked network. Compared to *tri*-PHV, *di*-PHV has fewer substituents, leading to a lower crosslinking density and *T_g_* in theory. However, the reality is that the *T_g_* values are numerically close, at 154.1 °C (*tri*-PHV-20) and 155.6 °C (*di*-PHV-20). The analysis of this phenomenon is consistent with the explanations obtained from DSC. Furthermore, the control had the highest crosslinking density and was free of dynamic imine bonds, resulting in the highest *T_g_* of about 187 °C.

TGA results show the thermal stability of the imine epoxy vitrimers (Figure 1e). Vitrimers with a 20% imine bond content were used as examples. The vitrimers produced were thermally stable, with a mass loss of less than 5% at 387 °C (Table 1). The initial decomposition temperature (defined as the temperature corresponding to a 5% mass loss, T_d5%_) of the control sample, which did not contain imine bonds, was slightly higher than that of imine epoxy vitrimers. This is due to the C=N structure of the imine bonds being less stable than the C-C bonds, leading to a lower initial degradation temperature. *R_800_* (the residual weight percentage at 800 °C) may also reflect the char yield or thermal degradation resistance at high temperatures. At 800 °C, all imine epoxy vitrimers showed higher carbon yields (over 32%) than the control sample (17%), which is ascribed to the easy char formation ability of imine bonds [36,37]. Meanwhile, the carbon yields of *di*-CHV-20, *di*-PHV-20, and *tri*-PHV-20 at 800 °C were 32.1%, 35.9% and 40.7%, respectively. Compared to *tri*-PHV-20 and *di*-PHV-20, which feature a rigid benzene ring structure, *di*-CHV-20 is more susceptible to thermal decomposition due to its flexible aliphatic chain, resulting in the lowest carbon yield. *Tri*-PHV has more substituents and thus a higher crosslinking density than *di*-PHV, which effectively slows down its thermal decomposition [44,52,53]. The results above indicate that the prepared bio-based imine epoxy vitrimers have good thermal stability and high carbon yields, giving our materials potential as flame-retardant materials. (Figure 1f).

The thermodynamic properties of imine-based vitrimers with varying crosslinking structures and imine bond contents are summarized in Table 1. Since the vitrimers with 10% imine bond contents do not allow adequate dynamic bond exchange above 230 °C (Figure S4) and the vitrimers with 30% imine bonds have poor thermodynamic properties, subsequent experiments were performed using vitrimers with 20% imine content.

### 3.3. Stress Relaxation of Bio-Based Imine Epoxy Vitrimers

**Figure 2:**
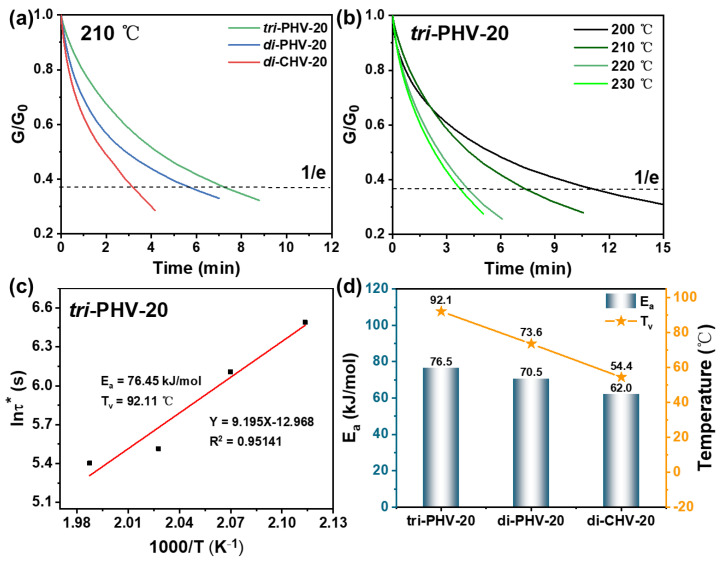
(**a**) Stress relaxation curves of *tri*-PHV-20, *di*-PHV-20, *di*-CHV-20 at 210 °C; (**b**) stress relaxation curves of *tri*-PHV-20 at temperatures of 200–230 °C; (**c**) linear fitting line of *tri*-PHV-20; (**d**) activation energy (*E_a_*) and topological freezing transition temperature (*T_v_*) of *tri*-PHV-20, *di*-PHV-20, *di*-CHV-20.

Stress relaxation is a characteristic feature of dynamic covalent polymers, enabling materials to be remodeled. Similarly, dynamic imine bonds can induce topological rearrangements in crosslinked networks through imine metathesis reactions. The time taken for the modulus to relax to 1/e (i.e., 0.37) of its initial value is defined as the relaxation time (τ*), as per the Maxwell model [52]. For the control and vitrimers with 10% imine bond content, barely any stress relaxation behavior can be observed (Figure S4). This is due to the low content of dynamic imine bonds, which was not sufficient to support the dynamics of the crosslinked network at a given temperature. In contrast, vitrimers with 30% imine bond content showed poor thermodynamic properties. Therefore, samples with a 20% imine bond content were selected for stress relaxation tests. Figure 2a shows that at a given temperature (210 °C), the τ* of the vitrimer in this work is *tri*-PHV-20 > *di*-PHV-20 > *di*-CHV-20. This can be attributed to the fact that *tri*-PHV-20 has a rigid benzene ring structure and a tri-substituent group, which restricts chain movement and hinders the exchange of dynamic imine bonds. *Di*-PHV-20 has only two substituent groups, showing the lower τ*. For *di*-CHV-20, the flexible aliphatic chain structure, rather than the rigid benzene ring structure, results in the strongest chain mobility and the lowest τ*.

In addition, test temperature affects the rate of imine exchange reactions. The relationship between stress relaxation and time from 200 to 230 °C (Figure 2b) demonstrates a significant decrease in τ* with increasing temperature. At lower temperatures, the rate of the imine exchange reaction is slow as the topological structure of vitrimer is frozen. High temperatures accelerate exchange reactions within the resin’s crosslinking network. According to the Arrhenius equation, the relationship between relaxation time and temperature is as follows [34,37]:

(1) $$ln\tau *=ln\tau_{0}+\frac{E_{a}}{RT}$$

where *τ*_0_ is the characteristic relaxation time at an infinite temperature, *E_a_* is the activation energy of the imine metathesis, and *R* is the universal gas constant. It is important to emphasize that the exchange activation energy (*E_a_*) measured through stress relaxation is essentially an apparent value, reflecting the sensitivity of the material’s viscosity to temperature changes rather than solely representing the intrinsic kinetics of the chemical exchange process. Consequently, the measured *E_a_* is not only determined by the inherent activation energy of bond exchange but is also significantly influenced by physical factors such as network topology and localized segmental rigidity. This implies that the observed stress relaxation time comprises both the characteristic timescale of bond exchange and the timescale of functional group diffusion within the network, with both factors collectively governing the overall relaxation behavior. The activation energy (*E_a_*) of the chain exchange is calculated by linear fitting between ln (τ*) and 1000/T according to the Arrhenius law. The topology freezing transition temperature (*T_v_*), which corresponds to the transition from a solid to a viscous fluid, is defined as the temperature at which a viscosity of 10^12^ Pa·s is reached. Hence, there is an equation with which to estimate the relaxation time of the network at *T_v_* [54,55]:

(2) $$\eta =\frac{1}{3}E^{\prime }\tau *$$

where *η* is the viscosity of the vitrimer at 10^12^ Pa·s and *E′* is the rubbery plateau modulus obtained from the DMA measurement. The Arrhenius-fitted line was then extrapolated to τ* to find the *T_v_*.

According to the curve fitting, the *E_a_* of *tri*-PHV-20, *di*-PHV-20, *di*-CHV-20 is 76.5 kJ/mol, 70.5 kJ/mol and 62.0 kJ/mol, and the *T_v_* is 92.1 °C, 73.6 °C and 54.4 °C, respectively (Figure S5 and Figure 2c,d). The trends in *E_a_* and *T_v_* are consistent with the analyses above. While the viscosity and relaxation time methods are commonly used for estimating *T_v_*, these approaches do come with inherent uncertainties, as highlighted in previous studies [56,57,58]. Thermal relaxation findings underscore the presence of dynamic properties in these bio-based imide vitrimers at these elevated temperatures.

### 3.4. Mechanical Properties of Bio-Based Imine Epoxy Vitrimers

**Figure 3:**
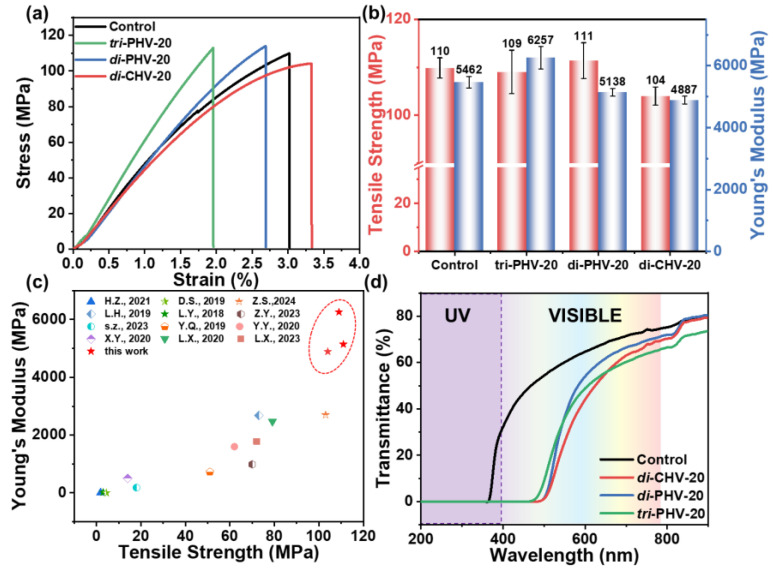
(**a**) Typical stress–strain curves and (**b**) the detailed mechanical properties for the control, *tri*-PHV, *di*-PHV and *di*-CHV; (**c**) comparison of tensile properties and Young’s modulus of *tri*-PHV, *di*-PHV and *di*-CHV with those of other imine vitrimers from the references [29,30,31,34,37,38,39,41,42,46,49,50]; (**d**) UV spectra of the control, *tri*-PHV, *di*-PHV and *di*-CHV.

The mechanical properties of the imine epoxy vitrimers were evaluated through tensile tests. Figure 3a shows the stress–strain curves, revealing the significant dependence of tensile properties on the molecular structures. As shown in Figure 3b and Table 2, for *tri*-PHV-20, the presence of a rigid benzene ring and its three substituents leads to high stiffness and high crosslinking density, thus exhibiting the highest Young’s modulus (6257 MPa) and lowest elongation (1.9%). For *di*-PHV-20, there are only two substituents on the benzene ring, resulting in a lower crosslinking density and Young’s modulus than that of *tri*-PHV. For *di*-CHV, there exists a large number of flexible aliphatic chain segments resulting in the lowest modulus (4887 MPa). Compared to *tri*-PHV, the control, despite having the highest crosslinking density, lacks the rigid C=N double bond and exhibits a lower Young’s modulus, whereas compared to *di*-PHV and *di*-CHV, the primary factor influencing the control of Young’s modulus is the crosslinking density, which results in a higher Young’s modulus (5462 MPa). Previous studies have shown that the modulus measured by UTM is typically higher than that obtained from DMA, with differences ranging from 1 to 3 or more times greater [42,49,50]. Moreover, due to their rigid crosslink structure and high crosslinking density, the tensile strengths of the control, *tri*-PHV, *di*-PHV and *di*-CHV are 110 MPa, 109 MPa, 111 MPa, 104 MPa, respectively. It is worth mentioning that the tensile properties of vitrimers herein prepared are much better than those of most bio-based imine epoxy vitrimers reported in the literature (Figure 3c), because epoxy vitrimers containing imine bonds tend to exhibit poor mechanical properties. Compared to those of most imine-based vitrimer materials, the highly branched topology and the multifunctional group design in this system allowed for a more complex and well-distributed network of crosslinking points, causing a denser three-dimensional crosslinked network. Moreover, the presence of aromatic structures imparts significant molecular rigidity, which restricts molecular chain mobility and enhances intermolecular π-π stacking interactions. These combined effects contribute to the increased modulus and strength of the material. It was discovered in this study that incorporating vanillin-derived monomers and amine curing agents effectively produces vitrimers with high moduli and high strengths. This approach capitalizes on the rigid aromatic imines and dense crosslinking, resulting in epoxy imine vitrimers with high strength.

### 3.5. UV-Shielding Properties of Bio-Based Imine Epoxy Vitrimers

Long-term exposure to ultraviolet light can fragment chemical bonds, hasten material aging and reduce service longevity. Therefore, improving the UV resistance of materials is crucial. Given the UV-absorbing capability inherent in the C=N structure within imine bonds, the results of UV-vis spectroscopy, conducted to gauge the transmittance of the control, *di*-CHV, *di*-PHV, and *tri*-PHV, were assessed. In the UV spectrum (200–400 nm), *di*-CHV, *di*-PHV, and *tri*-PHV exhibited 0% transmittance within the 200–480 nm range, indicating their effective blocking of UV light within the full UV spectrum (Figure 3d). By comparison, the control only showed 0% transmittance within the 200–360 nm range instead of the entire UV spectrum, indicating that the control sample can only block a portion of UV light. In the visible spectrum (400–780 nm), imine epoxy vitrimers showed lower transmission compared to the Control. This phenomenon is caused by the presence of imine groups in the vitrimer network, which act as chromophores that absorb UV light, resulting in the darker color of the vitrimers relative to that of the control. Generally speaking, the dynamic imine bonds in vitrimers demonstrate significant UV shielding capability. This material finds application in UV protection to mitigate UV-induced aging [36].

### 3.6. Degradability and Reprocessability of Bio-Based Imine Epoxy Vitrimers

Conventionally cured epoxy resins are difficult to recycle because of their permanent three-dimensional network. The dynamic imine bonds can undergo three reversible reactions including transamination, imine metathesis and imine hydrolysis, allowing the epoxy resin to be remolded and recycled at specific conditions. Among these, imine metathesis enables vitrimer reprocessability, while imine hydrolysis and transamination facilitate material degradability. Together, these mechanisms establish reprocessability through the physical pathway and degradation through the chemical pathway.

**Figure 4:**
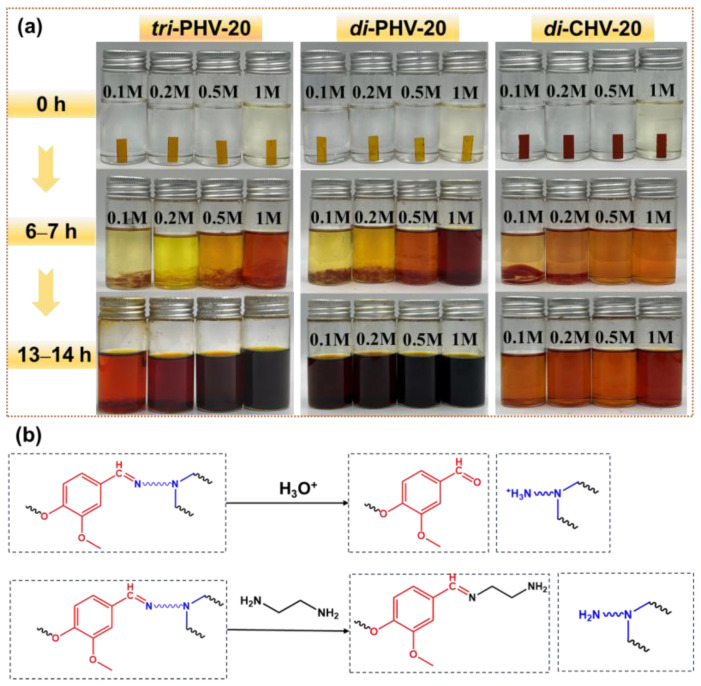
(**a**) Digital photo of degradation process of *tri*-PHV-20, *di*-PHV-20 and *di*-CHV-20 in *x* M HCl + EDA + DMSO (*x* = 0.1, 0.2, 0.5, 1, V_HCl_:V_EDA_:V_DMSO_ = 1:1:8); (**b**) degradation mechanism of vitrimers with HCl and EDA.

Under mildly acidic and amine-rich conditions, covalently crosslinked networks containing imine bonds undergo degradation, breaking down into small molecules with amino and aldehyde groups. HCl and EDA are degradation media that are commonly used to degrade imine epoxy vitrimers. As shown in Figure 4a, four concentrations of HCl (0.1 M, 0.2 M, 0.5 M, 1 M) were used, while the volume ratio of HCl, EDA and DMSO in the degradation solution was 1:1:8. The dissolution process of polymers typically involves two distinct stages: swelling and dissolution. Initially, solvent molecules penetrate the polymer network, leading to swelling. This facilitates the easier ingress of H^+^ ions and EDA into the crosslinked network, thereby hastening the polymer degradation process by bond exchange. The degradation mechanisms that occur via hydrolysis and transamination reactions are illustrated in Figure 4b. It was observed that *di*-CHV-20, with its flexible aliphatic chain, was completely degraded within 8 h, with the degradation rate reaching 3.3 mg/mL/h in 1 M HCl (Equation (S1)) [56,57]. *Di*-PHV-20 had reduced polymer network motility due to the introduction of the rigid benzene ring, which hindered the solvent molecules, EDA and H^+^, from entering the inside of the crosslinked network, leading to lower rate of degradation (1.3 mg/mL/h in 1 M HCl). Due to there being more substituents on the benzene ring, *tri*-PHV had the highest crosslinking density and slowest degradation rate (1.2 mg/mL/h in 1 M HCl) [50].

The degradation process of imine bonds is an acid-catalyzed reversible process, and the H^+^ concentration has a very significant effect on the degradation process of the imine epoxy vitrimers (Figure S6). As the concentration of the HCl solution increased (0.1 M, 0.2 M, 0.5 M, 1 M), the degradation rate of the imine epoxy vitrimers became higher, with the highest degradation rate in 1 M hydrochloric acid solution (*tri*-PHV: 1.2 mg/mL/h, *di*-PHV: 1.3 mg/mL/h, *di*-CHV: 3.3 mg/mL/h).

**Figure 5:**
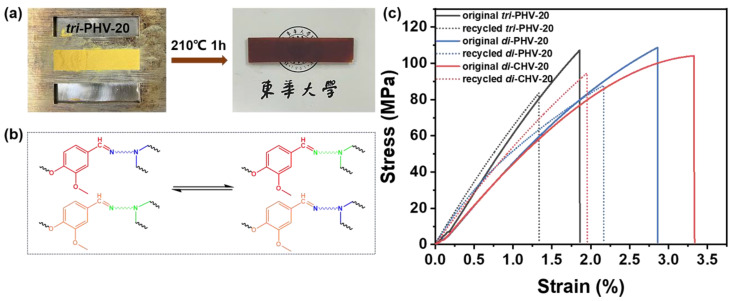
(**a**) Illustration of thermal reprocessing progress; (**b**) imine metathesis reaction of *tri*-PHV-20; (**c**) stress–strain curves for original and recycled *tri*-PHV-20, *di*-PHV-20 and *di*-CHV-20.

The dynamic imine bonds provide the imine epoxy vitrimers with excellent reprocessability. To investigate reprocessability, *tri*-PHV-20 was ground into small pieces and subsequently remolded using a hot press at 210 °C under 40 MPa for 1 h to ensure proper compaction (Figure 5a). After hot pressing, a new homogeneous resin block was obtained, which was the result of the sufficient bond exchanging reaction (Figure 5b). The recycled sample was subjected to tensile testing, and the results was compared with that of the original sample (Figure 5c and Table 2). After reprocessing, the elongation at break and tensile strength decreased and Young’s modulus increased. For example, the tensile strength, Young’s modulus and the elongation at break values of the reprocessed *tri*-PHV were 76%, 113% and 69% of those of the pristine sample, respectively. The reduction in tensile strength and elongation can be mainly attributed to the irreversible breakage of the permanent chemical bonds in the material system or aging during hot pressing [15,16,17]. The slight increase in Young’s modulus may have been due to the fact that the small amount of amino and aldehyde groups remaining in the original thermoset were post-cured after hot pressing, increasing the degree of crosslinking [23,36]. Briefly, the remolded resin still retained over 76% of its original tensile strength, indicating that incorporation of the dynamic imine bonds allows for excellent reprocessability and mechanical properties in epoxy without destroying its network structure.

### 3.7. Solvent Resistance and Gel Fraction of Bio-Based Imine Epoxy Vitrimers

To test the solvent resistance of the prepared vitrimers, the samples were immersed in DMSO, DMF, THF, ACE, EtOH, EAC, HCl, and NaOH solutions. The resin blocks were kept at room temperature for 7 days to observe the changes in mass and appearance. As can be seen in Figure S7, the resin blocks showed little swelling after 7 days of immersion in all solvents. In summary, the resin blocks remained physically unchanged and insoluble, and there was no change in the color of the solution, demonstrating excellent solvent resistance.

**Figure 6:**
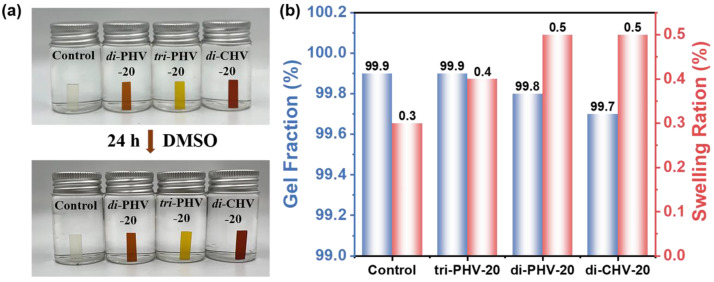
(**a**) Photographs of the control, *tri*-PHV-20, *di*-PHV-20 and *di*-CHV-20 samples dispersed in DMSO solution before and after 24 hat RT; (**b**) gel fraction and swelling ratio of the Control, *tri*-PHV-20, *di*-PHV-20 and *di*-CHV-20 in DMSO solution.

In the abovementioned solvent resistance test, it was possible to initially judge that DMSO underwent the highest degree of swelling. Therefore, DMSO was chosen as the solvent with which to further test the gel fraction of the obtained vitrimers. At room temperature, the control, *tri*-PHV-20, *di*-PHV-20, and *di*-CHV-20 were immersed in DMSO solution for 24 h for the swelling experiments (Figure 6a). The crosslinking effect was verified by investigating the gel fraction and swelling ratio of the bio-based imine vitrimer (Equation (S2)). As shown in Figure 6b, the gel fractions of all samples in DMSO solvents were above 99.7% or even higher, these fractions being comparable to those of thermosetting polymers with high crosslinking densities. The high gel fractions were due to the tightly packed crosslinked network, which prevented solvents from entering the interior of the network and reduced the dissolution of low-molecular-weight substances in the polymer network. At the same time, we found that the swelling ratios of the bio-based vitrimers in DMSO were all less than 0.5%. This indicates that the solvent molecules hardly penetrated into the polymer molecular network and did not interact or mix with the polymer segments, verifying the excellent crosslinking effect [43,59].

**Figure 7:**
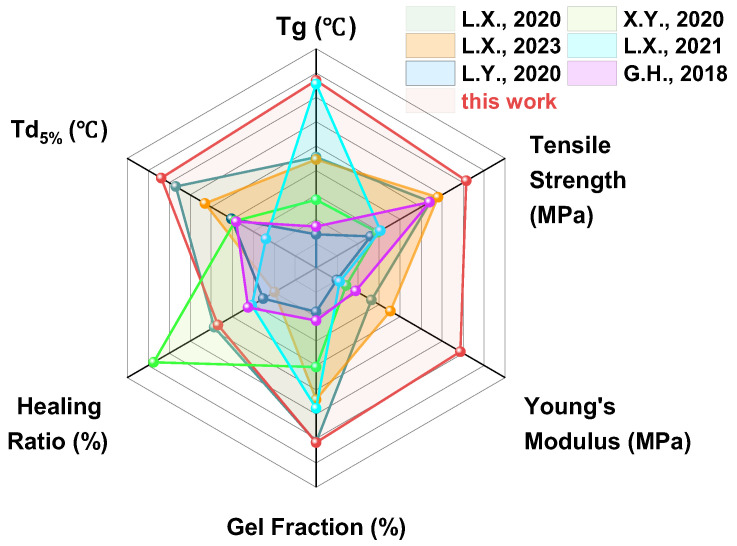
Comparison of overall performance of *tri*-PHV with that of other imine vitrimers from the references [42,44,46,49,50,52].

In brief, benefitting from their dense crosslinked network and rigid benzene ring structure, the samples in this work exhibited excellent thermal stability, mechanical properties, and chemical resistance, surpassing those of previously reported imine-based vitrimers (Figure 7).

## 4. Conclusions

Three bio-based imine epoxy vitrimers with dynamic imine bonds were synthesized using a solvent-free, one-pot method by simply mixing vanillin-derived aldehyde monomers, epoxy resins and conventional amine curing agents. Varying the dynamic crosslinked network structure allows for the physical mechanical properties of the vitrimers to be tailored. Specifically, *tri*-PHV had the best mechanical performance, with a 109 MPa tensile strength, Young’s modulus of 6256 MPa, and *T_g_*-DMA of 154 °C, which are higher than those of previously reported imine-based vitrimers. The dynamic imine covalent bonds enabled these crosslinked epoxy networks to be reprocessible and degradable. This study demonstrates a simple and effective process to prepare high-performance bio-based and recyclable epoxy thermosets. Our material exhibits significant potential for large-scale applications in structural composites and as a recyclable thermosetting material. Additionally, its bio-based composition further enhances its appeal as a sustainable alternative to conventional petroleum-derived thermosets, aligning with global efforts to develop eco-friendly and circular economy materials. Future work could also explore functional modifications, such as introducing self-healing properties, electrical conductivity, or flame retardancy, to expand their potential applications in advanced materials and smart coatings. This study provides valuable insights into the design of high-performance bio-based vitrimers, paving the way for next-generation recyclable thermosetting materials that combine mechanical strength, sustainability, and industrial feasibility.

## Figures and Tables

**Table 1:** The thermal properties, thermo-mechanical properties and thermal stability of bio-based imine epoxy vitrimers.

Sample	Imine Bond Content	*T_g_*_(DMA)_ (°C)	*T_g_*_(DSC)_ (°C)	*T*_d5%_ (°C) ^1^	*R*_800_ (%) ^2^
**Control**	/	187.0	173.2	402.0	17.5
***tri*-PHV**	10%	168.3	145.1	/	/
	20%	154.1	132.8	387.9	40.7
	30%	150.7	128.8	/	/
***di*-PHV**	10%	167.3	145.8	/	/
	20%	155.6	132.4	387.3	35.9
	30%	150.2	127.5	/	/
***di*-CHV**	10%	161.5	137.0	/	/
	20%	148.4	125.6	388.2	32.1
	30%	141.4	118.9	/	/

^1^ T_d5%_ is the temperature corresponding to a 5% mass loss. ^2^ R_800_ typically refers to the residual weight percentage at 800 °C, and the mathematical definition is R_800_ = (residual weight of the sample at 800 °C)/(initial weight of the sample) × 100%.

**Table 2:** The mechanical properties and recovery ratio of original and recycled bio-based imine epoxy vitrimers.

Sample	Tensile Strength (MPa)	Recovery ^1^ (%)	Young’s Modulus (MPa)	Recovery (%)	Elongation at Break (%)
**Control**	110	/	5462	/	3.0
**Original *tri*-PHV-20**	109	76	6257	113	1.9
**Recycled *tri*-PHV-20**	83		7068		1.3
**Original *di*-PHV-20**	111	78	5138	115	2.6
**Recycled *di*-PHV-20**	87		5886		2.2
**Original *di*-CHV-20**	104	89	4887	113	3.4
**Recycled *di*-CHV-20**	93		5498		1.9

^1^ The recovery is the ratio of the recycled mechanical properties to original mechanical properties.

## Data Availability

The original contributions presented in this study are included in the article and Supplementary Material. Further inquiries can be directed to the corresponding author.

## Supplementary Materials

The following supporting information can be downloaded at https://www.mdpi.com/article/10.3390/polym17050571/s1: Table S1: Vitrimers with varying epoxy and amine contents; Figure S1: ^1^H NMR spectra of (a) *di*-Ari, (b) *di*-Aro, and (c) *tri*-Aro; Figure S2: IR spectra of vitrimers of (a) aldehyde monomers, (b) BFDGE, DDS, *tri*-Aro, *tri*-PHV and (c) *tri*-PHV, *di*-PHV, *di*-CHV and the control; Figure S3: (a) *T_g_*-DSC and (b) *T_g_*-DMA of all bio-based imine expoxy vitrimers; Figure S4: Stress relaxation curves of (a) *tri*-PHV-10, (b) *di*-PHV-10, (c) *di*-CHV-10, and (d) the control at 230 °C; Figure S5: Stress relaxation curves according to the Arrhenius equation used to determine the activation energy of stress relaxation for (a) *tri*-PHV-20, (b) *di*-PHV-20, (c) and *di*-CHV-20 at temperatures of 200–230 °C, and linear fitting line of (d) *tri*-PHV-20, (e) *di*-PHV-20, and (f) *di*-CHV-20; Figure S6: (a) Degradation rate and (b) degradation time of the control, *tri*-PHV-20, *di*-PHV-20 and *di*-CHV-20 in *x* M HCl + EDA + DMSO solutions (*x* = 0.1, 0.2, 0.5, 1, V_HCl_:V_EDA_:V_DMSO_ = 1:1:8); Figure S7: Photographic images of imine in various solvents in the initial state and at 7 days.
