# Supplementary material for: Bio-Based and Solvent-Free Epoxy Vitrimers Based on Dynamic Imine Bonds with High Mechanical Performance
Source: Polymers (Basel). 2025 Feb 21;17(5):571. doi: 10.3390/polym17050571 (PMC11902475; doi:10.3390/polym17050571)
Supplement: Supplementary file 1 [file polymers-17-00571-s001.zip › polymers-3455912-supplementary.pdf]

polymers

MDPI

Supplementary Materials

Bio-Based and Solvent-Free Epoxy Vitrimers Based on Dynamic Imine Bonds with High Mechanical Performance

Lei Chen, Na Ning, Gang Zhou, Yan Li, Shicheng Feng, Zhengyan Guo and Yi Wei *

Center for Civil Aviation Composites, Donghua University, 2999 North Renmin Road, Shanghai 201620, China; chenlei2946@163.com (L.C.); foreverluck7@sina.com (N.N.); 1215042@mail.dhu.edu.cn (G.Z.); 1235040@mail.dhu.edu.cn (Y.L.); fsctcu@163.com (S.F.); zyguo712@163.com (Z.G.)
* Correspondence: weiy@dhu.edu.cn

Table S1. Vitrimers with varying epoxy and amine contents.

Sample	—CH(O)CH$_2$ (mol)	-CHO (mol)	-NH$_2$ (mol)
***di*-CHV-10**	0.36	0.04	0.12
***di*-CHV-20**	0.16	0.04	0.09
***di*-CHV-30**	0.093	0.04	0.22
***di*-PHV-10**	0.36	0.04	0.12
***di*-PHV-20**	0.16	0.04	0.09
***di*-PHV-30**	0.093	0.04	0.22
***tri*-PHV-10**	0.36	0.04	0.12
***tri*-PHV-20**	0.16	0.04	0.09
***tri*-PHV-30**	0.093	0.04	0.12

Characterization

Solvent resistance, gel fraction and degradation measurement. Solvent resistance of the obtained imine vitrimers was conducted by immersing strips of the control, *di*-CHV, *di*-PHV and *tri*-PHV in different solvents (DMSO, DMF, THF, ACE, EtOH, EAC, HCl, and NaOH solutions) for 7 days at room temperature.

The degradation was measured by placing samples under 130°C in 20 ml HCl and EDA solution (V_{HCl}:V_{EDA}:V_{DMSO}=1:1:8); the concentrations of HCl were 0.1M, 0.2M, 0.5M, and 1M. The degradation rate was measured by varying the hydrochloric acid concentration, and calculated according to Equation (S1):

$$R = \frac{m}{Vt} \tag{S1}$$

where *m* is the mass of the degraded sample, *V* is the volume of degraded liquid, and *t* is the time for complete degradation.

The swelling test was conducted by immersing sample stripes in DMSO for 24 h. The samples were removed and dried in an oven for 24 h. The gel fraction was calculated by Equation (S2):

$$GF = \frac{W_2}{W_1} \times 100\% \tag{S2}$$

where the initial weight is denoted as W_1 and the weight after drying is denoted as W_2.

The swelling ratio was calculated by Equation (S3):

$$SR = \frac{W_3 - W_1}{W_1} \tag{S3}$$

where W_1 is the initial dry mass of the sample, and W_3 is the swollen mass of the sample after immersion in the solvent for 24h.

$$Healing\ Ratio = \frac{Property\ of\ Healed\ Material}{roperty\ of\ Undamaged\ Material} * 100\% \quad (S4)$$

where "Property" can be any index characterizing the properties of the material, such as the mechanical strength, tensile strength, elongation at break, etc.

The chemical composition of the imine vitrimers was analyzed by Fourier transform infrared spectrometry (FTIR, VERTEX 70, BRUKER).

The spectral range of the measured light was 4000 ~ 400 cm^{-1}. The synthesis of bio-based imine vitrimers was characterized by ^1H NMR detection (AVANCE NEO, BRUKER) using $CDCl_3$ and DMSO as solvents.

Differential scanning calorimetry (DSC) analysis was performed by TA-Q2000 under a nitrogen (N_2) purge from 25 °C to 230 °C at a heating rate of 10°C/min.

The thermal decomposition in N_2 was measured by thermogravimetric analysis (TGA, 209 F3) at a heating rate of 20°C/min^{-1} from RT to 800°C.

Dynamic mechanical properties were measured by the dynamic mechanical analyzer (DMA, TA-Q800) at a heating rate of 3°C/min. The amplitude was set to 5 μm and the frequency was set to 1 Hz.

The tensile strength of the material was tested on a universal testing machine (ETM203BTS). Dog bone-shaped coupons were produced in metal die under hot pressing.

The UV spectra were recorded by a UV-vis-IR spectrophotometer (UV3600, Shimadzu) from 200 to 1000 cm^{-1}.

The stress relaxation measurement was performed on the DMA (TA-Q800). The samples were first heated to the test temperature and equilibrated with a stress of 0.001 N for 5 min; then, a constant strain of 1% was applied to the materials for stretching, and the change in stress with time was measured.

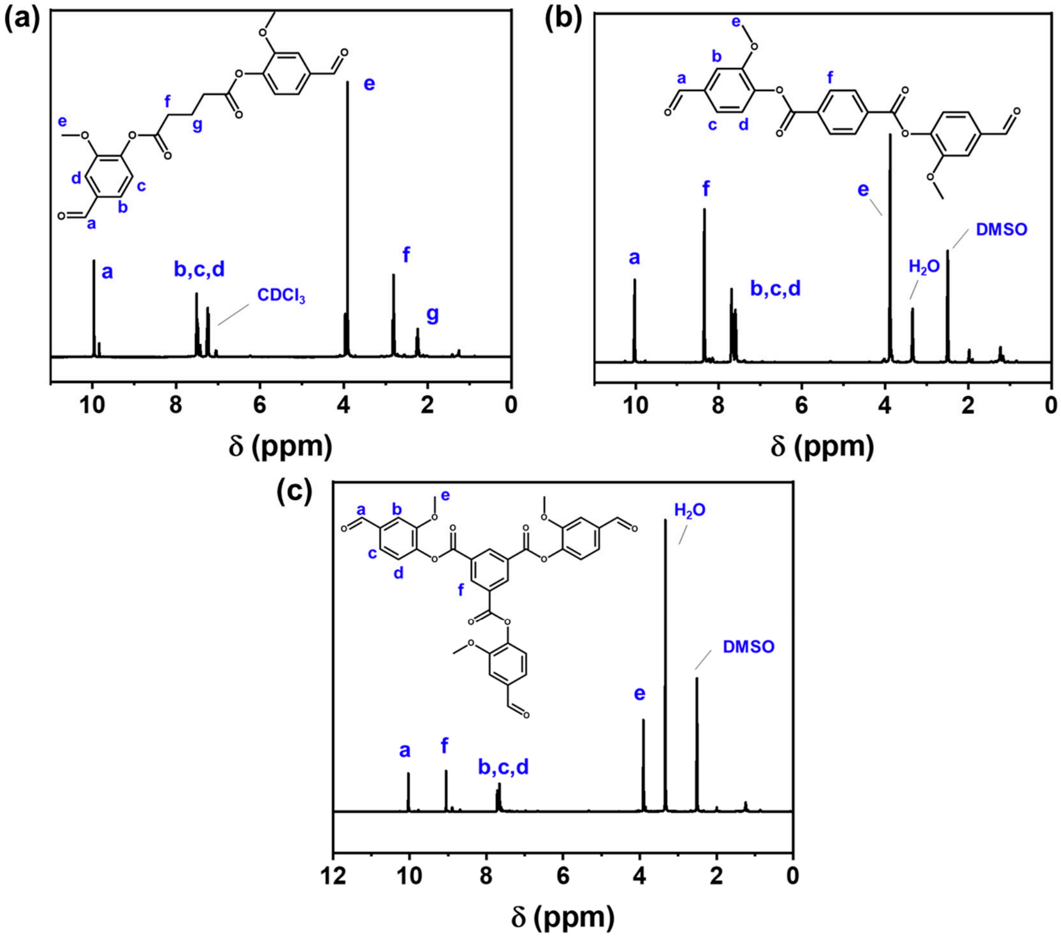

Figure S1. ^{1}H NMR spectra of (**a**) *di*-Ari, (**b**) *di*-Aro, (**c**) *tri*-Aro.

^{1}H-NMR (600 MHz, $CDCl_3$) *di*-Ari: δ(ppm) = 10(s, 2H, -CHO), 7.4-7.5(d, 6H, $-C_6H_6$), 3.9(s, 6H, $-OCH_3$), 2.9 (t, 4H, $-COCH_2-$), 2.1 (tt, 2H, $-CH_2-$).

^{1}H-NMR (600 MHz, DMSO) *di*-Aro: δ(ppm) = 10(s, 2H, -CHO), 8.3(d, 4H, $-C_6H_6$), 7.4-7.5(d, 6H, $-C_6H_6$), 3.9(s, 6H, $-OCH_3$).

^{1}H-NMR (600 MHz, DMSO) *tri*-Aro: δ(ppm) = 10(s, 3H, -CHO), 9(s, 3H, $-C_6H_6$), 7.7-7.6(d, 9H, $-C_6H_6$), 3.9(s, 6H, $-OCH_3$).

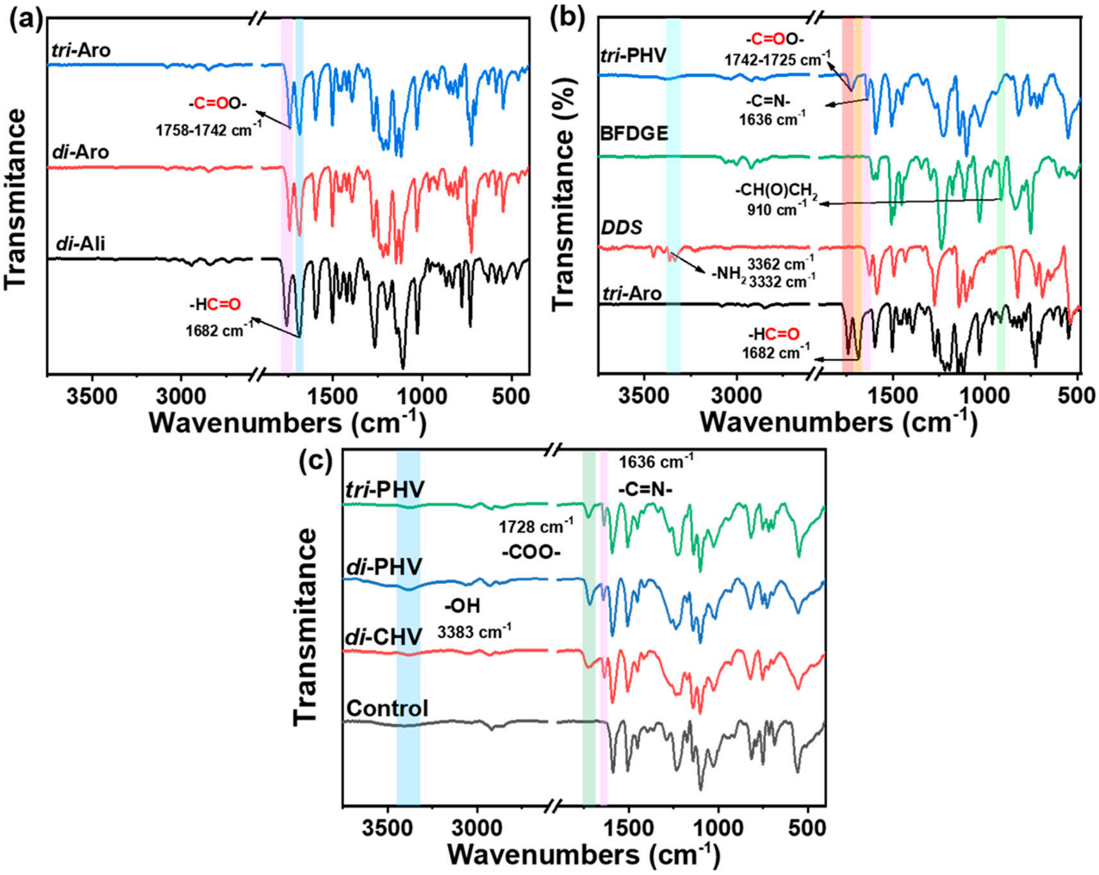

Figure S2. IR spectra of vitrimers of (**a**) aldehyde monomers, (**b**) BFDGE, DDS, *tri*-Aro, and *tri*-PHV and (**c**) *tri*-PHV, *di*-PHV, *di*-CHV and the control.

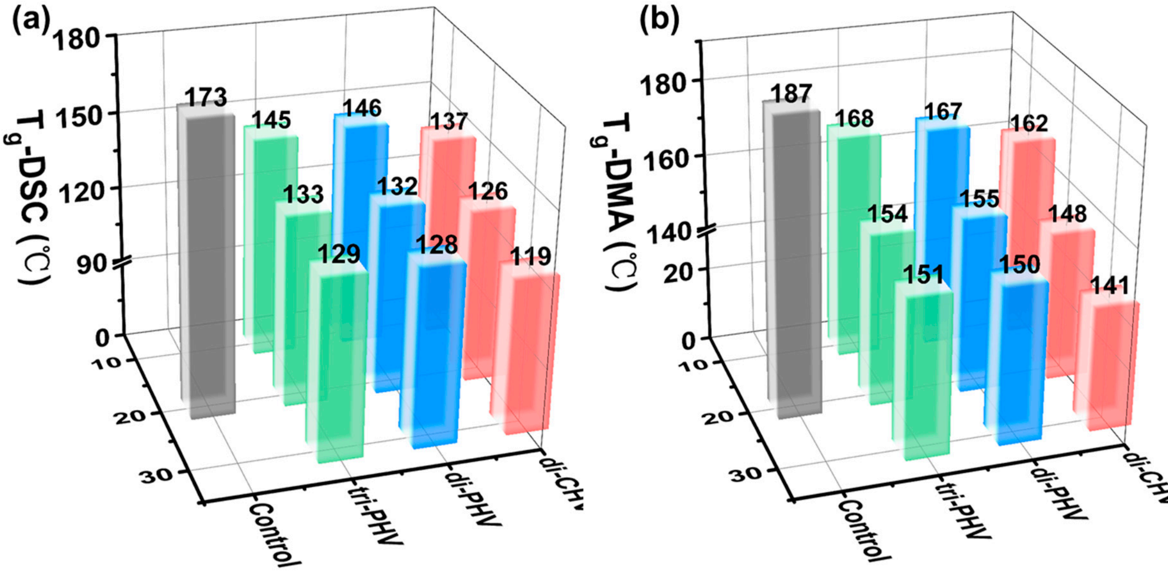

Figure S3. (a) T_g -DSC and (b) T_g-DMA of all bio-based imine epoxy vitrimers.

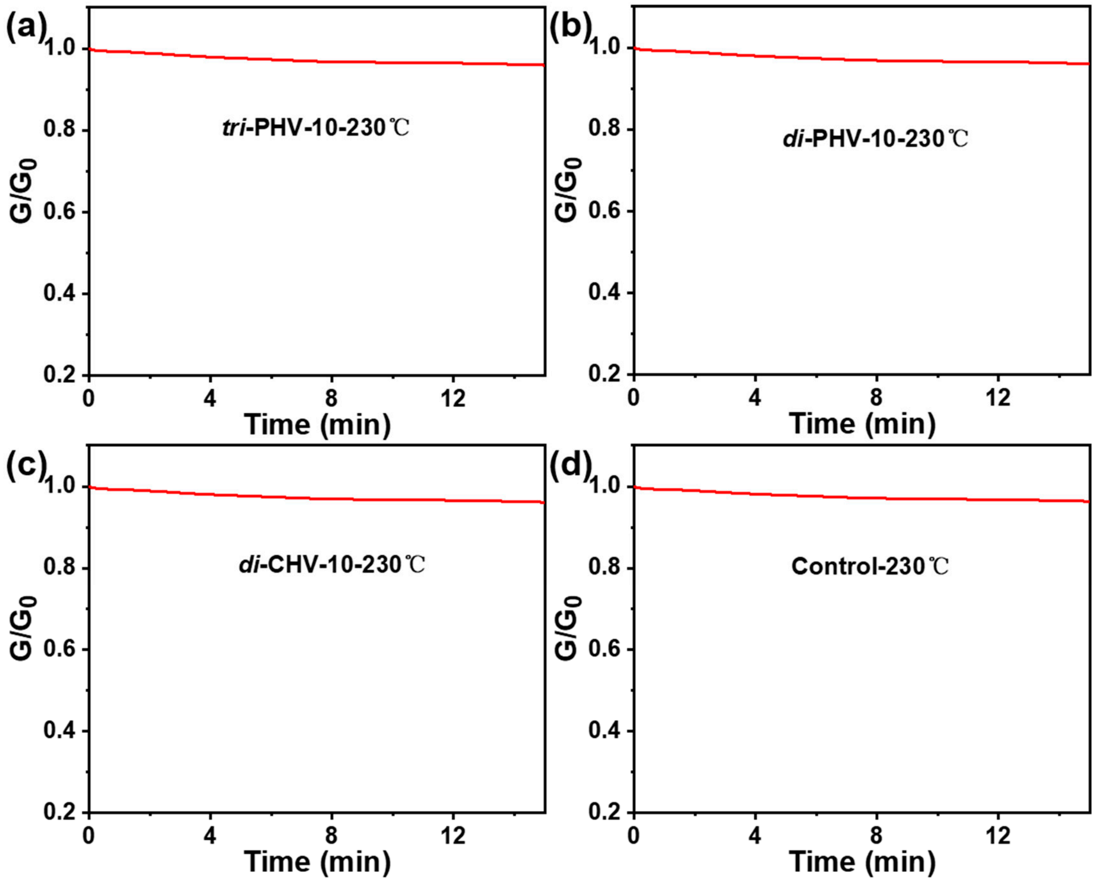

Figure S4. Stress relaxation curves of (**a**) *tri*-PHV-10, (**b**) *di*-PHV-10, (**c**) *di*-CHV-10, and (**d**) the control at 230°C.

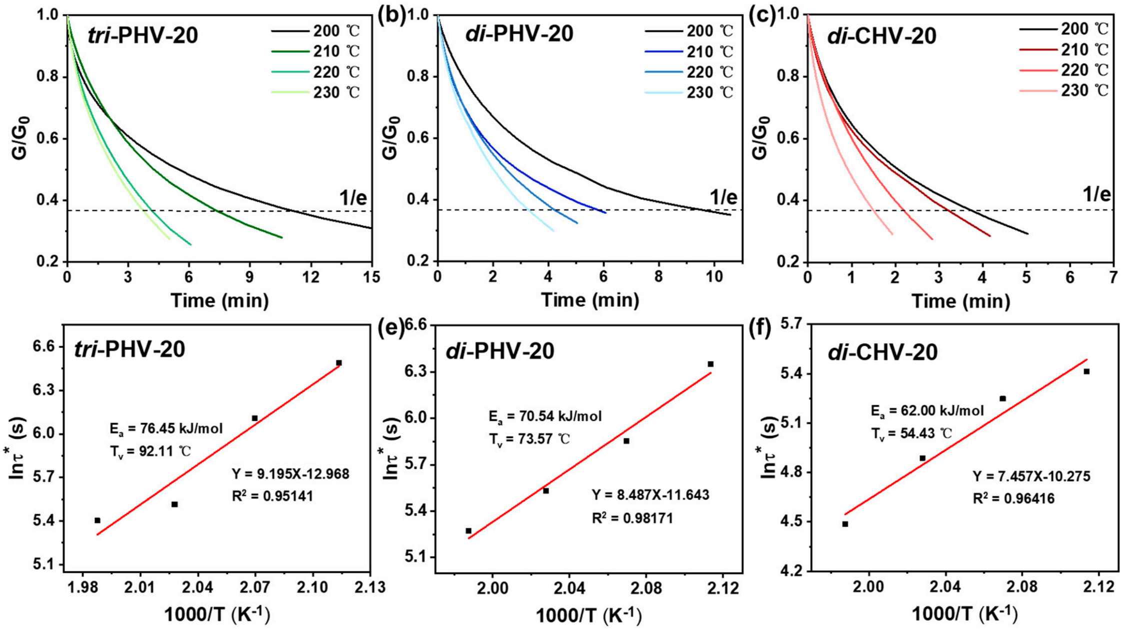

Figure S5. Stress relaxation curves according to the Arrhenius equation used to determine the activation energy of stress relaxation; (a) *tri*-PHV-20, (b) *di*-PHV-20, and (c) *di*-CHV-20 at temperatures of 200–230°C; linear fitting line of (d) *tri*-PHV-20, (e) *di*-PHV-20, and (f) *di*-CHV-20.

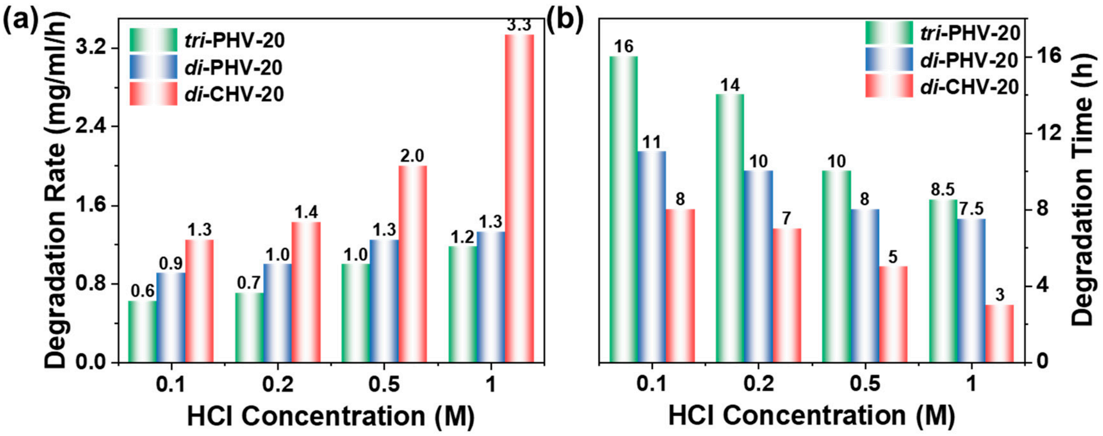

Figure S6. (a) Degradation rate and (b) degradation time of the control, *tri*-PHV-20, *di*-PHV-20 and *di*-CHV-20 in *x*M HCl + EDA + DMSO solutions (*x*=0.1, 0.2, 0.5, 1, V_{HCl}:V_{EDA}:V_{DMSO}=1:1:8).

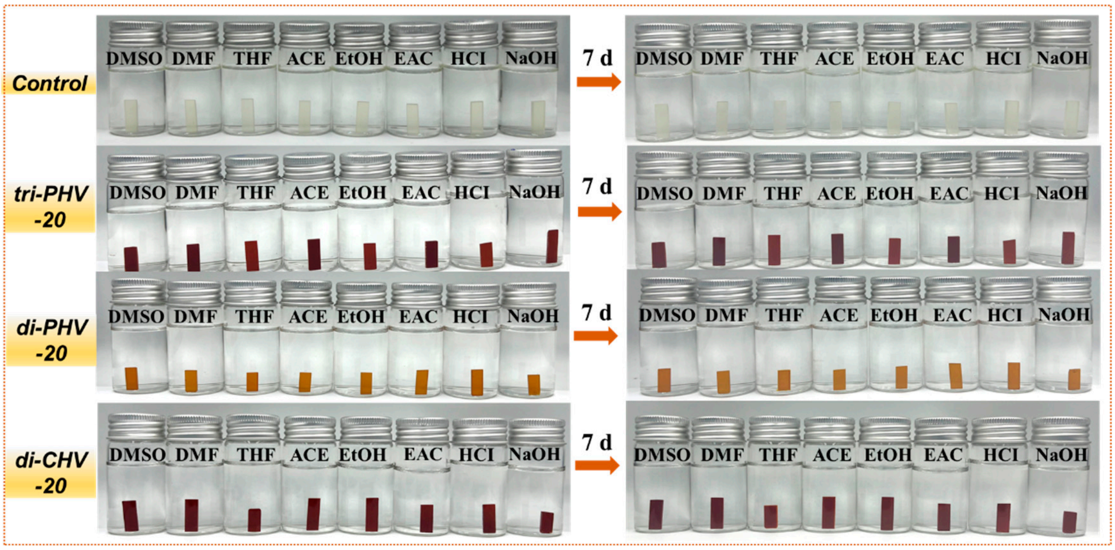

Figure S7. Photographic images of imine in various solvents in the initial state and 7 days.
